# Oxygen Uptake Kinetics Is Slower in Swimming Than Arm Cranking and Cycling during Heavy Intensity

**DOI:** 10.3389/fphys.2017.00639

**Published:** 2017-09-01

**Authors:** Ana Sousa, Fabio Borrani, Ferran A. Rodríguez, Grégoire P. Millet

**Affiliations:** ^1^Research Center for Sports, Exercise and Human Development, University of Trás-os-Montes and Alto Douro Vila Real, Portugal; ^2^Faculty of Biology and Medicine, ISSUL, Institute of Sport Sciences, University of Lausanne Lausanne, Switzerland; ^3^INEFC-Barcelona Sport Sciences Research Group, Institut Nacional d'Educació Física de Catalunya, University of Barcelona Barcelona, Spain

**Keywords:** exercise modes, triathletes, V̇O_2_ kinetics, gas exchange, modeling

## Abstract

Oxygen uptake ($\overset{\cdot }{\text{V}}{\text{O}}_{2}$) kinetics has been reported to be influenced by the activity mode. However, only few studies have compared $\overset{\cdot }{\text{V}}$O_2_ kinetics between activities in the same subjects in which they were equally trained. Therefore, this study compared the $\overset{\cdot }{\text{V}}$O_2_ kinetics response to swimming, arm cranking, and cycling within the same group of subjects within the heavy exercise intensity domain. Ten trained male triathletes (age 23.2 ± 4.5 years; height 180.8 ± 8.3 cm; weight 72.3 ± 6.6 kg) completed an incremental test to exhaustion and a 6-min heavy constant-load test in the three exercise modes in random order. Gas exchange was measured by a breath-by-breath analyzer and the on-transient $\overset{\cdot }{\text{V}}$O_2_ kinetics was modeled using bi-exponential functions. $\overset{\cdot }{\text{V}}$O_2peak_ was higher in cycling (65.6 ± 4.0 ml·kg^−1^·min^−1^) than in arm cranking or swimming (48.7 ± 8.0 and 53.0 ± 6.7 ml·kg^−1^·min^−1^; *P* < 0.01), but the $\overset{\cdot }{\text{V}}$O_2_ kinetics were slower in swimming (τ_1_ = 31.7 ± 6.2 s) than in arm cranking (19.3 ± 4.2 s; *P* = 0.001) and cycling (12.4 ± 3.7 s; *P* = 0.001). The amplitude of the primary component was lower in both arm cranking and swimming (21.9 ± 4.7 and 28.4 ± 5.1 ml·kg^−1^·min^−1^) compared with cycling (39.4 ± 4.1 ml·kg^−1^·min^−1^; *P* = 0.001). Although the gain of the primary component was higher in arm cranking compared with cycling (15.3 ± 4.2 and 10.7 ± 1.3 ml·min^−1^·W^−1^; *P* = 0.02), the slow component amplitude, in both absolute and relative terms, did not differ between exercise modes. The slower $\overset{\cdot }{\text{V}}$O_2_ kinetics during heavy-intensity swimming is exercise-mode dependent. Besides differences in muscle mass and greater type II muscle fibers recruitment, the horizontal position adopted and the involvement of trunk and lower-body stabilizing muscles could be additional mechanisms that explain the differences between exercise modalities.

## Introduction

Muscular exercise requires large changes in the metabolic rate often exceeding 10-fold the resting steady-state values (Poole and Jones, 2012). At the onset of constant moderate to heavy intensity exercise there is an early fast increase in oxygen consumption ($\overset{\cdot }{\text{V}}$O_2_), which is usually completed within the ~15–25 s of exercise (Phase I or cardio-dynamic phase). This early response is attributed to the increase in cardiac output and thus pulmonary blood, not reflecting the muscular $\overset{\cdot }{\text{V}}$O_2_ (Rossiter et al., 1999). After this initial phase, a rapid and exponential increase in $\overset{\cdot }{\text{V}}$O_2_, with a time constant between ~20–45 s, occurs (Phase II—primary or fast component), in which pulmonary $\overset{\cdot }{\text{V}}$O_2_ kinetics largely reflect the kinetics of O_2_ consumption in the exercising muscles (Grassi et al., 1996). This transitory phase, prior to the achievement of any steady state (moderate intensity) or prior to the development of the slow component phase (heavy and severe intensities), provides a window into the fundamental processes of muscle energetics and metabolic control that are otherwise not accessible (Poole and Jones, 2012).

The comparison of $\overset{\cdot }{\text{V}}$O_2_ kinetics between various sport activities, body posture, ergometers or groups has been used for providing insights to the fundamental mechanisms of the different phases (Poole and Jones, 2012). Among many facts the $\overset{\cdot }{\text{V}}$O_2_ kinetics are broadly altered when different ergometers are utilized, being the majority of studies in this field conducted by comparing running and cycling exercise. Aiming to explore the relationship between the $\overset{\cdot }{\text{V}}$O_2_ slow component ($\overset{\cdot }{\text{V}}$O_2sc_) and the muscle contraction regimen used, Carter et al. (2000) reported that the magnitude of the $\overset{\cdot }{\text{V}}$O_2sc_ phase was less for running than for cycling at heavy exercise. This could be related to differences in the muscle contraction regimen, which may have caused a relatively greater recruitment of the less efficient type II muscle fibers in cycling compared with running. At the severe intensity domain, Hill et al. (2003) reported that the time constant of the fast component phase was faster (reflecting the differences in the muscle contraction type involved) and its amplitude was greater (associated with higher oxygen demand) in running than in cycling. Moreover, the amplitude of the $\overset{\cdot }{\text{V}}$O_2sc_ phase was 40% smaller in running, concluding that the exercise modality affects the $\overset{\cdot }{\text{V}}$O_2_ kinetics even within the severe intensity. Extending the $\overset{\cdot }{\text{V}}$O_2_ kinetics analysis beyond the $\overset{\cdot }{\text{V}}$O_2sc_ phase with triathletes, Caputo and Denadai (2004) concluded that the time constant of the fast component phase during running and cycle exercise tests performed at maximal oxygen consumption ($\overset{\cdot }{\text{V}}$O_2max_) intensity were independent of the exercise mode performed, but dependent on the training status of the subjects These data suggest that the mechanical differences between cycling and running modes do not influence the time constant in the severe domain, contrarily to the $\overset{\cdot }{\text{V}}$O_2sc_.

Other hypothesis could explain differences in $\overset{\cdot }{\text{V}}$O_2_ kinetics during distinct exercise modes. The recruitment of a greater muscle mass could potentially compromise muscle perfusion (Saltin et al., 1998). Cycling engages the major muscle groups of the lower body, such that performance could be compromised compared with other exercise modes such as arm cranking, where a lower fraction of the total muscle mass is recruited (Drescher et al., 2015). In this latter exercise modality, the $\overset{\cdot }{\text{V}}$O_2sc_ can also be exercise mode-dependent due to the recruitment of type II muscle fibers, which are in higher proportions in the upper body (Bernasconi et al., 2006). Therefore, arm cranking exercise could be a valuable paradigm exercise to examine whether the additional type II muscle fibers recruitment could contribute to possible differences in $\overset{\cdot }{\text{V}}$O_2sc_.

Typically swimming requires also lower muscle mass (predominantly upper body) and induces lower maximal $\overset{\cdot }{\text{V}}$O_2_ and HR responses than cycling (Roels et al., 2005), which might be due to several additional factors such as different body position inducing greater hydrostatic pressure and lower perfusion in the capillary bed of the working muscle, resulting in a reduction in both blood flow and oxygen transport. Thus, the horizontal body position adopted by swimmers, with lower muscle perfusion pressure, presumably as a consequence of lower arterial pressure, may be a key difference between swimming and upright exercise modes (Koga et al., 1999), as suggested by the slower kinetics of ventilation and gas exchange during supine compared with upright cycling exercise (Hughson et al., 1991). Hence, this postural factor could influence peripheral oxygen extraction and $\overset{\cdot }{\text{V}}$O_2max_.

Although the influence of the mechanical differences between running and cycling on $\overset{\cdot }{\text{V}}$O_2_ kinetics are well known, few studies have compared the $\overset{\cdot }{\text{V}}$O_2_ kinetics within other exercise modes within the same group of athletes. Even fewer included intensities that were clearly within the heavy intensity domain. There are methodological constraints for comparing $\overset{\cdot }{\text{V}}$O_2_ kinetics in different exercise modes: it is well-known that faster $\overset{\cdot }{\text{V}}$O_2_ kinetics and reduced $\overset{\cdot }{\text{V}}$O_2sc_ amplitude are highly correlated with higher levels of aerobic fitness and improved training status (Koppo et al., 2004), whereas larger adaptations occur in the specific training mode —e.g., principle of specificity: swimming for swimmers (Roels et al., 2005). In other words, direct comparison between exercise modes might be valid only in subjects trained to the same extent in these tested modes. For these purposes, well-trained triathletes represent a unique and valuable group since they train indistinctly in swimming, cycling and running. This training pattern influences $\overset{\cdot }{\text{V}}$O_2max_ and other physiological responses in different exercise modes as an effect of cross-training transfer between the upper and lower body exercise modes (Millet et al., 2002).

It is presently unknown whether the different mechanical and physiological factors interplay in the three modes of exercise does influence the $\overset{\cdot }{\text{V}}$O_2_ kinetics within the heavy intensity domain, when the same group of athletes, trained to a similar extent in the different exercise modalities, are involved. The analysis of such influences would provide new insights into the underlying control mechanisms in each exercise mode. Therefore, the purpose of this study was to compare the on-$\overset{\cdot }{\text{V}}$O_2_ kinetic response during swimming, arm cranking and cycling within the same group of trained triathletes when exercising within the heavy intensity domain. It was hypothesized that the exercise mode would contribute to distinct on-transient $\overset{\cdot }{\text{V}}$O_2_ kinetic patterns.

## Materials and methods

### Participants

Ten well-trained male triathletes (age 23.2 ± 4.5 years; height 180.8 ± 8.3 cm; weight 72.3 ± 6.6 kg) participated in the study. The participants had a training background in triathlon for 7 ± 2 years, and underwent intensive training during 14 ± 4 h/week for at least 3 years. The study was approved by the by the institutional ethics committee of Montpellier (France) and conducted in accordance with the Declaration of Helsinki. All participants provided written informed consent before participation. They were familiarized with the incremental test procedure during training sessions performed prior to the testing, and were encouraged to give their best effort.

### Design

**Figure 1:**
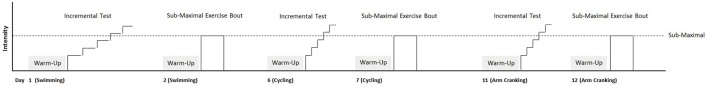
Schematic illustration of the testing protocols performed. See text for details.

The subjects completed three different maximal incremental exercise tests to assess maximal exercise capacity and cardiorespiratory parameters during free swimming, arm cranking, and cycling. Peak oxygen uptake ($\overset{\cdot }{\text{V}}$O_2peak_, ml·kg^−1^·min^−1^), ventilatory threshold (VT, % $\overset{\cdot }{\text{V}}$O_2peak_), peak power output (PPO, W) for cycling and arm cranking, and mean swimming velocity (v = d/t, m·s^−1^) were determined. Based on these parameters, workloads were calculated to eliciting equal relative intensity across the three exercise modes in a 6-min square-wave transition from rest to heavy-intensity for the assessment of on-transient $\overset{\cdot }{\text{V}}$O_2_ kinetic parameters. All tests were performed in the same conditions, that is, at the same time of day (±2 h) and with identical pre-test warm up: 10 min at the intensity corresponding to 50% of $\overset{\cdot }{\text{V}}$O_2peak_. The three different incremental tests were separated by a minimum of 72 h and completed in randomized order. The submaximal 6-min bouts mode were performed the day after the incremental test in the specific exercise mode. In the heavy-intensity bouts, parameters were estimated for the on-transient $\overset{\cdot }{\text{V}}$O_2_ kinetics and compared across the three exercise modes. Figure 1 shows a schematic of the testing protocol used.

#### Incremental tests

In swimming, the incremental test comprised five consecutive 200-m efforts at increasing speed with a 15-s rest interval (Bentley et al., 2005; Libicz et al., 2005). All tests involved in-water starts and open turns without underwater gliding, and took place in the same indoor 50-m pool. The speed of each swim was determined for each swimmer using personal best time (PB) in 400 m freestyle measured in the preceding month. The first 200-m stage was swum 30 s slower than the PB, and the three subsequent steps had to be completed 5 s slower per stage. The final swim was performed at maximal speed. The swimmer's speed was controlled using an Aquapacer “Solo” (Challenge and Response, Inverurie, UK) and subject's swimming pace was set by auditory signals at 12.5-m intervals, delineated by visual marks along the bottom of the pool. The incremental arm crank test was completed on an arm ergometer (Monark Exercise AB 881E, Monark-Crescent AB, Varberg, Sweden). The initial workload was 17.5 W and was increased by 17.5 W every minute until exhaustion. The incremental sitting cycle test was completed on an electromagnetic cycle ergometer (Ergoline, Bitz, Germany). The initial workload was 60 W and was increased by 30 W every minute until exhaustion. Criteria for exhaustion were: (i) heart rate (HR) attaining maximal theoretical HR (HR_max_ = 220 − age), and (ii) $\overset{\cdot }{\text{V}}$O_2_ leveling off even despite an increase in workload. PPO was defined as the highest mean external power output maintained during 1 min.

#### Sub-maximal 6-min exercise bouts

On subsequent days, the subjects performed 6-min of square-wave transition from rest to heavy intensity: constant workload at the metabolic intensity of 25%Δ above VT, i.e., calculated as VTΔ25% = [VT + 0.25 x ($\overset{\cdot }{\text{V}}$O_2peak_ − VT)].

### Physiological measurements

Gas exchanges during the three incremental tests were measured breath-by-breath (bxb) using a portable system (K4 b^2^, Cosmed, Rome, Italy). During the swimming tests, the gas analyzer was attached to a swimming snorkel (Aquatrainer, Cosmed, Rome, Italy) that has been previously validated (Rodríguez et al., 2008) and used to measure gas exchange during swimming. In the incremental tests, bxb data were reduced to 30-s bin averages and $\overset{\cdot }{\text{V}}$O_2peak_ was determined as the highest 30-s $\overset{\cdot }{\text{V}}$O_2_ average in either the swimming, arm cranking or cycling incremental tests. Using this protocol, the $\overset{\cdot }{\text{V}}$O_2peak_ was always attained at the final stage of the incremental test. VT was calculated using the v-slope method and coincided with $\overset{\cdot }{\text{V}}$O_2_ that elicited the first departure from linearity in $\overset{\cdot }{\text{V}}$ E/$\overset{\cdot }{\text{V}}$O_2_. VT was expressed as % $\overset{\cdot }{\text{V}}$O_2peak_ in each exercise mode.

In the sub-maximal 6-min bouts, bxb data were first examined to exclude from the analysis the values greater than 3 SD from the local mean. Then data of the two square-wave transitions from rest to heavy-intensity were interpolated into 1-s values. In order to enhance the reliability in determining the parameters describing the $\overset{\cdot }{\text{V}}$O_2_ kinetics, data were filtered through a 4th Butterworth low-pass digital filter with a cut-off frequency equal to 0.05 Hz (Robergs et al., 2010). To remove the influence of the cardiodynamic phase on the subsequent response we chose to exclude the first 20 s of data from the analysis. The on-transient $\overset{\cdot }{\text{V}}$O_2_ kinetics was modeled according to the Equation (1):

(1) $$\dot{\text{V}}{\text{O}}_{2}(t)=\left\{\begin{array}{cc} \dot{\text{V}}{\text{O}}_{\text{2base}} & \text{fort}<{\text{td}}_{\text{p}}\\ \dot{\text{V}}{\text{O}}_{\text{2base}}+{\text{A}}_{\text{p}}(1-{\text{e}}^{-({\text{t}-\text{td}}_{\text{p}})/\tau_{\text{p}}}) & \text{for }{\text{td}}_{\text{p}}\leq \text{t}<{\text{td}}_{\text{sc }}\;(\text{primary component})\\ \dot{\text{V}}{\text{O}}_{\text{2base}}+{\text{A}}_{\text{p}}(1-{\text{e}}^{-({\text{td}}_{\text{sc}}-{\text{td}}_{\text{p}})/\tau_{\text{p}}})+{\text{A}}_{\text{sc}}(1-{\text{e}}^{-(\text{t}-{\text{td}}_{\text{sc}})/\tau_{\text{sc}}}) & \text{for t}\geq {\text{td}}_{\text{sc}}\;(\text{slow component}) \end{array}\right\}$$

where $\overset{\cdot }{\text{V}}$O_2_ (t) represents the weight-related $\overset{\cdot }{\text{V}}$O_2_ at a given time t; $\overset{\cdot }{\text{V}}$O_2base_ is the baseline, resting $\overset{\cdot }{\text{V}}$O_2_ (taken as the first 30-s average $\overset{\cdot }{\text{V}}$O_2_ of the last minute before the start of exercise); td_p_, τ_p_, A_p_ represent the time delay, the time constant and the amplitude of the primary component, respectively; and td_sc_, τ_sc_, A_sc_, represent the equivalent parameters for the slow component. A_p_ was used to determine the gain (G_p_ = A_p_/P) of the primary component in cycling and arm cranking exercise.

Because the asymptotic value of the second function is not necessarily reached at the end of the exercise, the amplitude of the $\overset{\cdot }{\text{V}}$O_2_ slow component was defined as:

(2) $${\text{A}}_{\text{sc}}^{\prime }={\text{A}}_{\text{sc}}\left(\text{1}-{\text{e}}^{-\left(\text{te}-{\text{td}}_{\text{sc}}\right)/\tau_{\text{sc}}}\right)$$

where te was the time at the end of the exercise bout.

To estimate $\overset{\cdot }{\text{V}}$O_2_ kinetics parameters, equations were fitted to the exercise data (Microsoft Excel Solver) using an iterative procedure (Generalized Reduced Gradient), by minimizing the sum of the mean squares of the differences between modeled and measured $\overset{\cdot }{\text{V}}$O_2_ values. The relative contribution of the slow component to the net increase in $\overset{\cdot }{\text{V}}$O_2_ (%A_sc_') was calculated as the ratio between the amplitude of A_sc_' and the total increase of $\overset{\cdot }{\text{V}}$O_2_ during exercise. Effort intensity perception was rated recorded using the Borg 6–20 RPE Scale during all maximal incremental tests.

### Statistical analysis

All values are reported as mean ± standard deviation (SD). After analysis of normality (Kolmogorov-Smirnov test) and homogeneity of variance (Levene test) of the data, analysis of variance with repeated measures (RM-ANOVA) was used to compare the values from the three incremental tests (swimming, arm cranking, and cycling), as well as the $\overset{\cdot }{\text{V}}$O_2_ kinetics parameters obtained from the three square-wave transition from rest to heavy-intensity exercise. Significant main effects were subsequently analyzed using the Student-Newman-Keuls *post-hoc* test. Before the analysis, significance level was set at 5%.

The bootstrap method was used in the present study to assess the coefficient of variation (CV%) of the model parameters. It consists in resampling the original data set with replacement to create a number of “bootstrap replicate” data sets of the same size as the original data set. A random number generator to determine which data of the original data set will be included in a replicate data set was used. This was repeated 1,000 times, and the parameters estimated were retained. The CV of these 1,000 replicates was calculated.

## Results

### Maximal incremental exercise

The maximal workload reached at exhaustion was 353 ± 42 W in cycling, 142 ± 22 W in arm cranking, and 1.214 ± 0.064 m·s^−1^ in swimming. Both weight-related and absolute $\overset{\cdot }{\text{V}}$O_2peak_ were higher in cycling (65.6 ± 4.0 ml·kg^−1^·min^−1^ and 4.7 ± 0.6 l.min^−1^) than in arm cranking (48.7 ± 8.0 ml·kg^−1^·min^−1^ and 3.4 ± 0.4 l.min^−1^; *P* < 0.001, 0.007, respectively) and swimming (53.0 ± 6.7 ml·kg^−1^·min^−1^ and 3.8 ± 0.3 l.min^−1^; *P* < 0.001, 0.024, respectively). With the exception of weight-related $\overset{\cdot }{\text{V}}$O_2peak_ arm cranking-swimming (*r* = 0.77, *P* = 0.026), no correlations were found for cycling-arm cranking and cycling-swimming (*r* = 0.40 and 0.36; *P* = 0.24 and 0.31). Absolute $\overset{\cdot }{\text{V}}$O_2peak_ values (*r* = −0.12, 0.25, and 0.23; *P* = 0.77, 0.48 and 0.23, respectively) for cycling-arm cranking, cycling-swimming, and arm cranking-swimming were not correlated. No differences were found in maximal HR values (188 ± 7, 179 ± 8 and 175 ± 9 bpm; *P* > 0.05) or RPE (18.3 ± 0.6, 18.6 ± 0.6 and 17.3 ± 0.9 a.u.; *P* > 0.05), for cycling, arm cranking and swimming, respectively.

### $\overset{\cdot }{\text{V}}$O_2_ kinetics during heavy-intensity exercise

The average workload during the submaximal 6-min bouts was 266 ± 37 W in cycling, 98 ± 18 W in arm cranking, and 1.044 ± 0.048 m·s^−1^ in swimming, corresponding to 75 ± 2, 70 ± 6, and 86 ± 4 percent of maximal workload (but to 76 ± 2, 69 ± 6, and 71 ± 4 percent of $\overset{\cdot }{\text{V}}$O_2peak_) for cycling, arm cranking and swimming, respectively. $\overset{\cdot }{\text{V}}$O_2peak_ attained in these submaximal tests was higher in cycling (55.2 ± 5.9 ml·kg^−1^·min^−1^) than in arm cranking, or swimming (31.9 ± 5.6 and 41.5 ± 5.5 ml·kg^−1^·min^−1^, respectively; *P* < 0.001 and 0.001 respectively), but values were not correlated (*r* = 0.40, 0.36, and 0.25 for cycling-arm cranking, cycling-swimming, and arm cranking-swimming, *P* = 0.40, 0.36, and 0.29, respectively).

**Figure 2:**
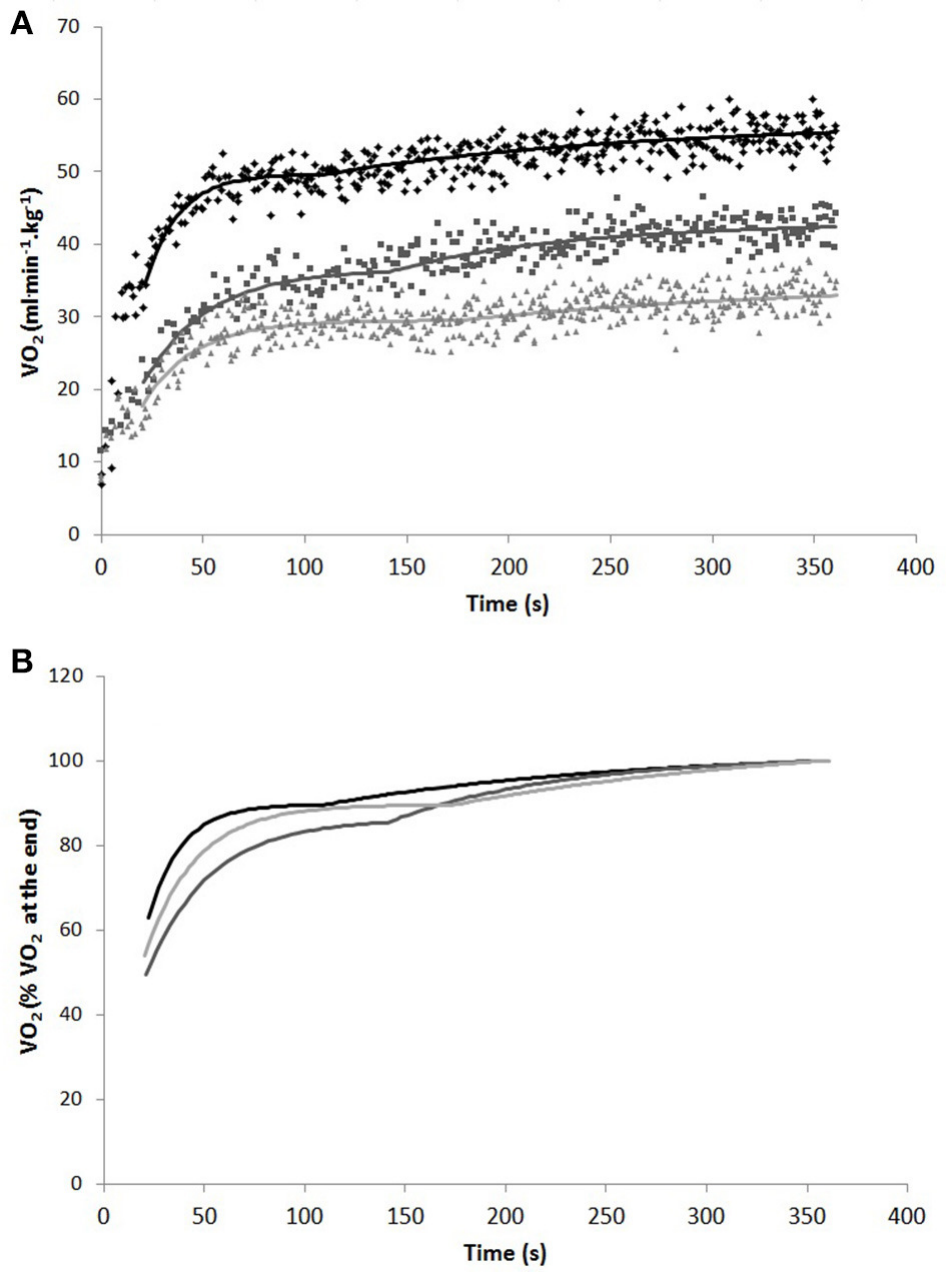
Representative breath-by-breath and best fit $\overset{\cdot }{\text{V}}$O_2_ kinetics curves in weight-related $\overset{\cdot }{\text{V}}$O_2_ values **(A)** and best fit $\overset{\cdot }{\text{V}}$O_2_ kinetics curves in percentage of $\overset{\cdot }{\text{V}}$O_2_ at the end of the exercise **(B)** in one subject during cycling (black), swimming (dark gray), and arm cranking (light gray). To remove the influence of the cardiodynamic phase on the subsequent response, the first 20 s of data were excluded from the analysis.

Figure 2 shows representative breath-by-breath and best-fit $\overset{\cdot }{\text{V}}$O_2_ kinetics curves for the three exercise modes, expressed both in weight-related $\overset{\cdot }{\text{V}}$O_2_ values (A), and in percentage of $\overset{\cdot }{\text{V}}$O_2_ at the end of the exercise (B).

Table 1 shows the estimated $\overset{\cdot }{\text{V}}$O_2_ kinetic parameters and the associated CV (%) during the 6-min square-wave transition from rest to heavy-intensity for all modes of exercise.

**Table 1:** Values for the estimated $\overset{\cdot }{\text{V}}$O_2_ kinetic parameters in the 6-min square-wave transition from rest to heavy-intensity (to Δ25%) for swimming, arm cranking and cycling).

	**Swimming**	**Arm cranking**	**Cycling**
$\overset{\cdot }{\text{V}}$O_2base_ (ml·kg^−1^·min^−1^)	8.1 ± 1.7	7.1 ± 1.2	8.1 ± 0.8
	(20.4)	(17.1)	(10.2)
A_p_ (ml·kg^−1^·min^−1^)	28.4 ± 5.1^*^^#^	21.9 ± 4.7^*^	39.4 ± 4.1^#^
	(17.9)	(21.5)	(10.3)
G_p_ (ml·min^−1^·W^−1^)	–	15.3 ± 4.2^*^	10.7 ± 1.3
		(27.5)	(12.2)
τ_p_ (s)	31.7 ± 6.2^*^^#^	19.3 ± 4.2^*^	12.4 ± 3.7^#^
	(19.7)	(21.9)	(22.0)
A_sc_' (ml·kg^−1^·min^−1^)	4.5 ± 1.8	3.8 ± 1.2	5.4 ± 1.4
	(39.9)	(32.3)	(25.5)
%A_sc_ (%)	13.4 ± 3.9	9.5 ± 5.3	13.7 ± 2.8
	(29.0)	(56.4)	(20.6)

*Data are mean ± SD (CV%). CV%, mean coefficient of variation in %; $\overset{\cdot }{V}$O_2base_, resting VO_2_; A_p_ and A_sc_', amplitude of the primary and slow components, respectively; τ_p_, time constant of the primary component; G_p_, gain of the primary component (G_p_ = A_p_/P); %A_sc_', % contribution of slow component to net increase in $\overset{\cdot }{V}$O_2_*.

* P < 0.05 differences with cycling;

# *P < 0.05 for differences with arm cranking*.

$\overset{\cdot }{\text{V}}$O_2base_ was not different between modes of exercise, but A_p_ was lower in both arm cranking and swimming compared with cycling (*P* < 0.001, for both), but higher in swimming compared with arm cranking (*P* < 0.005). Contrarily, gain was higher in arm cranking compared with cycling (*P* < 0.05). During swimming, subjects exhibited slower $\overset{\cdot }{\text{V}}$O_2_ kinetics compared with arm cranking (*P* < 0.05) and cycling (*P* < 0.001), but faster during cycling compared with arm cranking (*P* < 0.05). The A_sc_ and A_sc_' values were not different among exercise modes.

## Discussion

This study compared the on-transient $\overset{\cdot }{\text{V}}$O_2_ kinetics response during swimming, arm cranking and cycling in 6-min square-wave transition from rest to heavy-intensity. The novelty of our approach lies in that the same group of triathletes—whose training involves swimming, cycling and running, and thus the musculature of all body segments— exercised in the three modalities at comparable levels of metabolic intensity (i.e., 25% between VT and $\overset{\cdot }{\text{V}}$O_2peak_). Albeit exercising at a similar relative workload and for an equal duration, (i) the $\overset{\cdot }{\text{V}}$O_2_ kinetics was faster in cycling, followed by arm cranking and swimming; (ii) the amplitude of the fast component was largest in cycling, followed by swimming and arm cranking; and (iii) the gain of the primary component was greater in arm cranking compared with cycling.

### $\overset{\cdot }{\text{V}}$O_**2peak**_ and related parameters

The mean $\overset{\cdot }{\text{V}}$O_2peak_ values observed are in accordance with data reported previously for swimmers (Rodríguez, 2000), however higher than healthy subjects familiarized with arm cranking exercise (Bernasconi et al., 2006). As would be expected, $\overset{\cdot }{\text{V}}$O_2peak_ in cycling was 26 and 19% higher than in arm cranking and swimming, respectively, in line with previous studies. Sawka (1986), in a review of 18 studies, reported an average $\overset{\cdot }{\text{V}}$O_2peak_ during arm cranking corresponding to ~22% of $\overset{\cdot }{\text{V}}$O_2peak_ during cycling. Two recent studies with well-trained triathletes also reported average $\overset{\cdot }{\text{V}}$O_2peak_ values during cycling that were 22% (Roels et al., 2005) and 36% (Barrero et al., 2014) higher compared with those in swimming. The smaller skeletal muscle mass involved during (mostly but not exclusively) upper body exercise is likely to be the main contributor to this ~20–35% lower $\overset{\cdot }{\text{V}}$O_2peak_. Consequently, cardiorespiratory responses to swimming exercise will be influenced, as well, by other factors, such as HR_max_ and $\overset{\cdot }{\text{V}}$_E_ (Roels et al., 2005), which have been related with lower $\overset{\cdot }{\text{V}}$O_2peak_ measured in triathletes while swimming compared with cycling (Roels et al., 2005; Barrero et al., 2014) and running (Barrero et al., 2014). Although HR_max_ was not significantly different in our study, a trend for lower values in swimming (~175 bpm) and arm cranking (~179 bpm) compared with cycling (~188 bpm) was observed. Interestingly, in a study comparing trained swimmers and triathletes, the former exhibited 12% higher $\overset{\cdot }{\text{V}}$O_2peak_ in swimming than in cycling, whereas the opposite was found in the triathletes (22% higher $\overset{\cdot }{\text{V}}$O_2peak_ in cycling), highlighting the relevance of specific training (Roels et al., 2005). Therefore, both central and peripheral factors seem to interplay in determining the $\overset{\cdot }{\text{V}}$O_2peak_ attained during maximal exercise. Not withstanding the similar relative intensity performed during the heavy-intensity bouts, the subjects were also capable of performing well in the different exercise modes, and were similarly well trained in cycling and swimming;, therefore, the absence of differences in-between their $\overset{\cdot }{\text{V}}$O_2peak_ (~76, 69, and 71% in cycling, arm cranking and swimming, respectively) were observed.

### $\overset{\cdot }{\text{V}}$O_2_ primary component response

Within the heavy intensity exercise domain, τ_p_ reflects the rate at which the $\overset{\cdot }{\text{V}}$O_2_ response achieves the steady state before the appearance of the “excess” $\overset{\cdot }{\text{V}}$O_2_ of the slow component phase. Previous studies have reported a slower adjustment of $\overset{\cdot }{\text{V}}$O_2_ in arm exercise compared with leg exercise occurs, either when expressed as the half-time for the initial adaptation of $\overset{\cdot }{\text{V}}$O_2_ at exercise onset (Cerretelli et al., 1977), or as the mean response time for the overall response (Koga et al., 1996; Koppo et al., 2002), as long as the power output does equally elevate blood lactate (Casaburi et al., 1992). This difference in the kinetics was corroborated with the present results; i.e., arm cranking and swimming showing a substantially slower adjustment in $\overset{\cdot }{\text{V}}$O_2_, i.e. higher τ_p_, compared with cycling exercise (~19 and 32 vs. ~12 s, respectively). When comparing highly trained subjects of four sport modalities exercising in a time-to-exhaustion test at 100% of $\overset{\cdot }{\text{V}}$O_2peak_, Sousa et al. (2015) also found slower $\overset{\cdot }{\text{V}}$O_2_ kinetics for swimming (~21 s) compared with cycling (~16 s), rowing (~12 s), and running (~10 s). The faster rate of $\overset{\cdot }{\text{V}}$O_2_ increase compared with the present study can be attributed to the dissimilar intensity and duration of the exercise (i.e., ~3.7 min at 100% $\overset{\cdot }{\text{V}}$O_2max_ vs. 6 min of heavy intensity exercise). Similarly, Rodríguez et al. (2015), in a recent study with swimmers comparing 100-m all-out arm stroke, leg kick and whole stroke swims, showed also slower $\overset{\cdot }{\text{V}}$O_2_ kinetics in arm exercise (~12 s) compared with whole stroke (~9 s) and leg kicking exercise (~10 s). Testing physical education students who practiced a variety of sports, including those that predominantly utilize the upper body muscles, Koppo et al. (2002) also showed slower $\overset{\cdot }{\text{V}}$O_2_ kinetics for arm cranking exercise, compared with 6-min high intensity cycling exercise. Therefore, this study extends previous findings by corroborating that the $\overset{\cdot }{\text{V}}$O_2_ kinetics pattern in-between arm and leg exercises is different, even with trained triathletes performing well in these exercise modes—and not only with physically active or highly trained subjects not specifically and concomitantly trained in these modes—are tested.

This new feature of our study may help to explain the possible mechanisms responsible for the slower $\overset{\cdot }{\text{V}}$O_2_ kinetics in arm exercise. It was previously suggested by Casaburi et al. (1992) and Koga et al. (1996) that such a slower response during arm exercise could be due to the lower training status of the upper-body muscles compared with the lower-body muscles, since the untrained subjects tested in both studies could have had a higher conditioning status in these than those of the upper limbs. Collectively, this and the above cited studies suggest that the “training status” of the subject's musculature seems not to be, or at least, not to a great extent, determinant in the slower $\overset{\cdot }{\text{V}}$O_2_ kinetics of the upper vs. the lower body exercise at heavy intensity. However, it should be taking into account that although the present study was conducted with triathletes capable of performing well in different modes of exercise, arm cranking involves a distinct muscle recruitment pattern. This latter could, in some extend, reflect a different movement efficiency, and therefore, influence $\overset{\cdot }{\text{V}}$O_2_ kinetics.

Another possible explanation for the modality dependence of $\overset{\cdot }{\text{V}}$O_2_ slower kinetics could rely on the skeletal muscle fiber type found in the upper and lower body. On the one hand, this is supported by the fact that upper-arm muscles contain generally a higher proportion of type II fibers —e.g., in tennis players (Sanchis-Moysi et al., 2010) or cross-country skiers (Mygind, 1995)—, the proportions of type II fibers was shown to be higher in the *triceps brachii* compared to *vastus lateralis* muscles. On the other hand, it has also been reported that the percentage of type I fibers in the *gastrocnemius, vastus lateralis*, and *deltoid* was high in all, but not different among muscles, while the muscle respiratory capacity ($\overset{\cdot }{\text{Q}}$O_2_) and mean citrate synthase activity of the deltoid were lower than the *gastrocnemius* (Flynn et al., 1987). Type II fibers are metabolically less efficient, and not only the time constant of $\overset{\cdot }{\text{V}}$O_2_ primary component is significantly longer in upper-body muscles with a predominance of type II fibers (Kushmerick et al., 1992), but also is longer in subjects with a high proportion of type II fibers in the *vastus lateralis* (Pringle et al., 2002). Muraki et al. (2004), by studying *triceps brachii* muscle oxygen saturation using NIRS during arm cranking and cycling exercise in young women, noted a faster increase in the respiratory exchange ratio and a lower VT in arm compared with leg exercise, suggesting accelerated anaerobic glycolysis. They also concluded that the oxidative capacity of the arm muscles was limited due to early O_2_ utilization rather than poor O_2_ supply to the exercising musculature. The influence of fiber types composition and $\overset{\cdot }{\text{V}}$O_2_ primary component was previously addressed by Doria et al. (2011) in an elegant study, where the authors showed that a prolonged and active sojourn in hypoxia promoted an increase in the expression of slow isoforms of both heavy and light myosin subunits of vastus lateralis muscle in mountaineers. Moreover, this shift in muscle fiber type was accompanied by a decrease in mean response time of $\overset{\cdot }{\text{V}}$O_2_ kinetics at the onset of step submaximal cycling exercise. Therefore, in swimming exercise, the larger recruitment of type II muscle fibers could also have contributed to the slower $\overset{\cdot }{\text{V}}$O_2_ kinetics observed.

The fiber type controlling mechanism was also previously investigated within animal models. In a study with rats, muscles comprised of a high proportion of type II fibers have been shown to optimally increase their fractional O_2_ extraction during intensive exercise compared with predominantly slow-twitch muscles, which may be important for ensuring high blood-myocyte O_2_ flux and, therefore, a greater oxidative contribution to energetic requirements (McDonough et al., 2005). Moreover, for the same power output, “steady-state” $\overset{\cdot }{\text{V}}$O_2_ was reported to be larger in arm than in leg exercise, implying the mechanical efficiency to be lower during arm exercise (Vokac et al., 1975). Of interest are the reports that *in vitro* $\overset{\cdot }{\text{V}}$O_2_ on-kinetics of single muscle fibers at high intensity differ between high and intermediate oxidative muscle fiber with high oxidative muscle fibers yielding shorter time constant values than intermediate muscle fibers (30 vs. 50 s, respectively) during stimulated isometric contractions (Wüst et al., 2013). This difference seems to be in the same order of magnitude as that shown between arm cranking and cycling in the present study. Although one cannot infer full-body exercise responses from results on isolated fiber, this indirectly supports the speculation that fiber type differences underlie the differences in fast component between the different exercise modes. Collectively, these studies point out that one likely explanation for the slower $\overset{\cdot }{\text{V}}$O_2_ kinetics in arm cranking and swimming heavy intensity exercise, compared with cycling, is the greater recruitment of type II muscle fibers in the upper-body musculature.

Another potential explanation for the slower rate of $\overset{\cdot }{\text{V}}$O_2_ increase during swimming is related to the horizontal body position adopted during exercise. In the supine position, the blood flow to the working leg muscles is reduced, presumably as a consequence of lower arterial pressure in the legs when the effect of gravity (i.e., hydrostatic gradient effect) is removed (Koga et al., 1999). The same authors, Koga et al. (1999) reported that heavy exercise in the supine position was associated with a reduced amplitude of the fast component, which may be due, at least in part, to an attenuated early rise in HR in the supine position. The resultant fall in perfusion pressure to the active muscles leads to reduced exercise tolerance and slower $\overset{\cdot }{\text{V}}$O_2_ kinetics compared to upright exercise (Hughson et al., 1991). Results from MacDonald et al. (1998), who found that slower $\overset{\cdot }{\text{V}}$O_2_ kinetics at the onset of knee extension and flexion exercise in the supine compared with the upright position was accompanied by a slower increase of lower femoral artery blood flow, seem to support this hypothesis. In addition, the inability to produce maximal muscle contractions due to environmental constraints —i.e., body posture and propulsion is achieved by applying forces in a fluid medium— could also have limited the rate of increase in $\overset{\cdot }{\text{V}}$O_2_ during swimming (Sousa et al., 2015). Other possible contributing mechanisms could be the involvement of static muscle actions for trunk and leg stabilization and differences in the muscular action regime (i.e., concentric vs. eccentric exercise), whether by themselves or related to fiber type recruitment or decreased muscle perfusion to the working muscles (see previous discussion).

Other differences were found in the fast component response: (i) the amplitude in cycling was ~37% larger than in swimming and ~50% greater than in arm cranking, which is proportional to the greater $\overset{\cdot }{\text{V}}$O_2peak_ in cycling compared with swimming (~20%) and arm cranking (~25%); and (ii) the gain was higher in arm cranking (~15 ml·min^−1^·W^−1^) compared with cycling (~11 ml·min^−1^·W^−1^). This latter trend corroborates previous findings for high intensity exercise, although the values reported in our study are slightly higher (Koppo et al., 2002). These findings are consistent with the hypothesis of a greater recruitment of type II fibers during arm cranking and swimming than during cycling exercise. In fact, Bernasconi et al. (2006) documented an increase in the electromyography signal of *biceps brachii, triceps brachii, anterior deltoid*, and *infraspinatus* muscles during heavy arm cranking exercise. In addition, Pringle et al. (2002) reported that G_p_ during heavy cycle exercise was positively correlated with the percentage of type I fibers in the *vastus lateralis*. Therefore, differences in fiber type composition between the muscles of the upper and lower body play an important role in the fast component gain.

### $\overset{\cdot }{\text{V}}$O_2_ slow component response

No significant differences were found in the $\overset{\cdot }{\text{V}}$O_2sc_ amplitude (absolute and relative) or the time constant in-between modes of exercise, which does not corroborate previous reports conducted in cycling and arm cranking exercise (Koga et al., 1999). Multiple putative mechanisms of the $\overset{\cdot }{\text{V}}$O_2sc_ phenomenon have been postulated (Poole and Jones, 2012), the discussion of which exceeds the scope of this paper. However, in a recent work where a computer model of the skeletal muscle bioenergetic system was used, Korzeniewski and Zoladz (2015) postulated that the generation of $\overset{\cdot }{\text{V}}$O_2sc_ during heavy exercise could be related with: (i) the progressive inhibition of anaerobic glycolysis by accumulating protons (together with a slow decrease of the net creatine kinase reaction rate), (ii) the gradual increase of ATP usage during exercise, and (iii) perhaps, a decrease in the P/O ratio (ATP molecules/O_2_ molecules). It follows that a greater recruitment of type II muscle fibers would theoretically have an effect on $\overset{\cdot }{\text{V}}$O_2sc_. Koga et al. (1999) reported that heavy exercise in the supine position was associated with a greater amplitude of $\overset{\cdot }{\text{V}}$O_2sc_ but also, concomitantly, with a reduced amplitude of the fast component; this latter effect may be due, at least in part, to an attenuated early rise in HR in the supine position. These and other data seem to indicate that the amplitudes of the primary and slow components are sensitive to changes in muscle blood flow, which may influence, in turn, motor unit recruitment patterns. However, assuming that our subjects were equally well trained in all modes of exercise, we interpreted our results to indicate that either an equivalent anaerobic energy contribution to muscle metabolism, or that a similar progressive loss of muscle contractile efficiency—associated with the development of fatigue—occurred in the three exercise modalities, not measurably affecting the amplitude of the fast or slow components.

A limitation of the present study should be acknowledged, as the athletes performed only one transition to heavy exercise in each of the three modalities. Despite the number of transitions necessary for an adequate confidence in model parameter estimates is not clearly defined, it should be recognized that three (Spencer et al., 2011) or more exercise transitions might have improved such confidence. However, in endurance trained subjects, the number of repetitions may be reduced due to higher $\overset{\cdot }{\text{V}}$O_2max_ and lactate threshold, as suggested by Koga et al. (2005). Moreover, since we did not perform muscle biopsies on the subjects, it is impossible to determine the exact fiber type composition or phenotype of the arm vs. legs muscles. We agree that knowing the fiber type distribution and the oxidative capacity (e.g., mitochondrial content/efficiency, CS activity) would help clarifying some above-speculated mechanistic differences between the three activities modes used in this study.

In conclusion, this study provides further evidence of meaningful differences in the $\overset{\cdot }{\text{V}}$O_2_ kinetics across exercise modalities, noted in the same group of athletes similarly trained in-between them. Specifically, (i) $\overset{\cdot }{\text{V}}$O_2peak_ in cycling was ~26 and ~19% higher than in arm cranking and swimming, respectively, mainly attributable to the smaller skeletal muscle mass involved during upper body exercise; (ii) albeit the same trained athletes performed heavy intensity cycling, arm cranking and swimming exercises at a similar relative intensity, substantially slower $\overset{\cdot }{\text{V}}$O_2_ kinetics in swimming (~31 s) and arm cranking (~19 s) compared with cycling exercise (~12 s) was observed; (iii) also the amplitude of the primary component in cycling was ~37% larger than in swimming and ~50% greater than in arm cranking, in proportion to the greater $\overset{\cdot }{\text{V}}$O_2peak_ in cycling compared with swimming, although muscular efficiency (i.e., G_p_) was higher in arm cranking compared with cycling; (iv) although the slow component was evident in all exercise modes, unlike in previous studies, no significant differences were noted in the amplitude of the slow component (absolute and relative); and (v) overall, it is suggested that the muscle mass involved, the greater and/or faster recruitment of type II muscle fibers during upper body exercise, the dynamics of fatigue onset, the horizontal position adopted during swimming, differences in muscle perfusion, and the involvement of trunk and lower-body stabilizing muscles are plausible explanations for the differences found in the $\overset{\cdot }{\text{V}}$O_2_ kinetics patterns during heavy exercise, independently of the “training status” of the subjects.

## Author contributions

FB and GM contributed to the conception or design of the work and the acquisition of data. AS and FR contributed to data analysis and interpretation. AS, FB, FR, and GM contributed to the drafting of the manuscript or revising it critically for important intellectual content. AS, FB, FR, and GM contributed to the final approval of the version to be published. AS, FB, FR, and GM contributed to the agreement to be accountable for all aspects of the work in ensuring that questions related to the accuracy or integrity of any part of the work are appropriately investigated and resolved.

### Conflict of interest statement

The authors declare that the research was conducted in the absence of any commercial or financial relationships that could be construed as a potential conflict of interest.
